# The Impact of Genetic Relationship and Linkage Disequilibrium on Genomic Selection

**DOI:** 10.1371/journal.pone.0132379

**Published:** 2015-07-06

**Authors:** Hongjun Liu, Huangkai Zhou, Yongsheng Wu, Xiao Li, Jing Zhao, Tao Zuo, Xuan Zhang, Yongzhong Zhang, Sisi Liu, Yaou Shen, Haijian Lin, Zhiming Zhang, Kaijian Huang, Thomas Lübberstedt, Guangtang Pan

**Affiliations:** 1 Maize Research Institute of Sichuan Agricultural University, Chengdu, China; 2 Guangzhou Genedenovo Biotechnology Co., Ltd, Guangzhou, China; 3 Guangxi Maize Research Institute, Guangxi Academy of Agricultural Sciences, Nanning, China; 4 Institute of Plant Protection, Sichuan Academy of Agricultural Sciences, Chengdu, China; 5 Department of Agronomy, Iowa State University, Ames, IA, United States of America; 6 Interdepartmental genetics program, Iowa State University, Ames, IA, United States of America; University of Guelph, CANADA

## Abstract

Genomic selection is a promising research area due to its practical application in breeding. In this study, impact of realized genetic relationship and linkage disequilibrium (LD) on marker density and training population size required was investigated and their impact on practical application was further discussed. This study is based on experimental data of two populations derived from the same two founder lines (B73, Mo17). Two populations were genotyped with different marker sets at different density: IBM Syn4 and IBM Syn10. A high-density marker set in Syn10 was imputed into the Syn4 population with low marker density. Seven different prediction scenarios were carried out with a random regression best linear unbiased prediction (RR-BLUP) model. The result showed that the closer the real genetic relationship between training and validation population, the fewer markers were required to reach a good prediction accuracy. Taken the short-term cost for consideration, relationship information is more valuable than LD information. Meanwhile, the result indicated that accuracies based on high LD between QTL and markers were more stable over generations, thus LD information would provide more robust prediction capacity in practical applications.

## Introduction

With rapid development of high density genotyping technologies, such as SNP (single nucleotide polymorphism) arrays and GBS (genotyping by sequencing) [1–3], markers covering genomes at high density become available for application in plant and animal breeding. Traditional marker-assisted selection (MAS) focuses on markers, which are significantly associated with traits of interest. MAS has been shown to bias breeding value estimates [4,5]. This limitation can be overcome by genomic selection (GS), a promising approach for improving quantitative traits [6], which has been widely used in animal breeding [7]. In GS schemes, marker effects are estimated in a training population. Ideally, this training population is genetically closely related to the breeding population. Using the training population, both marker genotypes and trait phenotypes are combined in a statistical model. Effects of all markers are simultaneously estimated without statistical threshold. Breeding values of individuals related to the training population can then be predicted based on genomic estimated breeding values (GEBVs) from genotyping data only, using the marker effects estimated from the training population.

With high density genotypic data, there are not enough degrees of freedom to fit all marker effects to a data set of limited trait observations by least squares [5]. To overcome this limitation, several statistical models have been developed, including Bayesian shrinkage regression [6,8], random regression best linear unbiased prediction RR-BLUP [6,9], kernel regression [10] and machine learning methods [11,12]. Statistical models have been examined using empirical data of cattle [13], barley, maize, wheat, and Arabidopsis [14,15]. In most cases, RR-BLUP showed a robust prediction accuracy for different genetic architectures, especially when marker density was low to medium.

Accuracy of GS depends on the characteristics of the training population and validation population, such as heritability of traits [16], extent of linkage disequilibrium (LD), genetic distance [17], and genetic relationship between two populations [18,19]. Expected accuracy depends on marker density, effective sample size, and the number of included phenotypes [16]. Marker density greatly affects the costs of genotyping and is, therefore, a key factor in large scale application of GS [20]. Marker number had very little effect on prediction accuracies within families from various plant species, if marker densities were not very low (hundreds of markers with interval of 8~25cM for most traits) [14]. High marker coverage (thousands of markers or more) was needed, when GS was applied in a more diverse population [21]. In bi-parental populations, prediction accuracy is mostly due to genetic relationship captured by markers that contribute most to trait expression. However, if the genetic relationship between individuals is weak in a diverse population, dense coverage of markers is required to capture most of the markers in LD with QTL. The effect of these two factors for accuracy of GS were addressed in a simulation study [22], but empirical data in plants have not yet been reported.

Here, we used empirical data to study the contribution of genetic relationship and LD on prediction accuracy of GS under different scenarios due to different population structures, marker densities, and progeny numbers. Our objectives were, to (1) study the impact of LD on the number of markers and progenies required to obtain acceptable prediction accuracy within or across populations, and (2) investigate the effect of genetic relationship and LD on prediction accuracy of GS.

## Materials and Methods

### Genotypic and phenotypic data

Data from two maize (*Zea mays* L.) populations, IBM Syn10 [23] and IBM Syn4 [24], were used for this study (hereafter referred to as Syn10 and Syn4). In short, Syn10 is a B73×Mo17 doubled haploid (DH) population obtained after 10 generations of intermating, and Syn4 is a B73×Mo17 recombinant inbred line population obtained after four generations of intermating. Both Syn4 and Syn10 populations were planted in separate but adjacent experiments at the Agronomy Agricultural Engineering Research Center, Ames, Iowa, in 2006 and 2007. Performance *per se* was tested for each line. Growing degree days (GDD) were calculated in °C day from planting until the date, when at least 50% of the tassels in the plots were shedding pollen. Plant height (PH) was measured from soil surface to the flag leaf collar on five representative plants within each plot. For each experiment, both populations were repeated twice in a row column alpha lattice experimental design of seven columns and 37 rows. The two parents B73 and Mo17 were planted in a randomized fashion eight times within replications in each experiment, respectively. Each plot size consisted of 5.3 x 1.5 m², at a density of 69,187 plants/ha. 15 days prior to planting in May, 175 kg urea/ha were applied, as well as Metolachlor and Atrazine herbicides at a rate of 1.86 and 1.12 kg of active ingredient per ha. Neither herbicides nor insecticides were applied on the experiments after planting.

Genotype data for a set of 244 Syn4 recombinant inbred lines (RILs) are available at MaizeGDB [25], with 1339 polymorphic markers covering an approximately 6,240 cM (CentiMorgan) linkage map. A set of 194 Syn10 DH lines was genotyped using a genotyping by low coverage whole genome sequencing procedure [26]. Briefly, the sequencing reads from DH lines were aligned to the B73 reference genome. The genotype of each genomic region in 100 kb increments was determined by the proportion of reads derived from both parents, and adjacent regions with completely identical genotype in each line were integrated as bin-marker. A high-density genetic map of Syn10 consists of 6611 bin markers, with a genetic distance of 11198.5 cM (unpublished data).

### Imputation, LD, and kinship measures

Missing marker genotypes of Syn4 were imputed using a function in the package *R/QTL* that uses a hidden Markov model to predict missing marker genotypes, given observed multipoint marker data [27]. Considering convenience of subsequent comparisons between Syn4 and Syn10, genotypes from Syn10 were imputed onto Syn4 by first determining the physical position of the two markers sets in the B73 reference genome (B73 RefGen_v2) [28]. Then, for each Syn10 marker, the unknown Syn4 genotype values were imputed based on the nearest flanking markers in a given Syn4 line, similar to the method used to impute missing marker values in Syn4. Finally, all 6611 bin markers from Syn10 were imputed into Syn4 and used for further analyses.

The degree of LD between markers was quantified using the parameter *r* ^*2*^ [29], estimated using *GOLD* software [30]. LD within populations (Syn4, Syn10) and across populations was measured. Average LD was calculated in increments of 100 kb, according to marker distances. The realized relationship (kinship) matrix of two populations was calculated using TASSEL software [31] with imputed genotype data.

### BLUP of marker breeding values

As the aim of our project was to predict breeding values (BV), we used models to fit only the additive effects at each marker. The method used for BV prediction in this study was RR-BLUP [6]. The phenotypic values for a set of progenies were modeled as *y* = *μl* + *Xg* +*e*, where *y* is an *N*_*p*_ × *l* vector of phenotypic means of the progenies; *l* is an *N*_*p*_ × *l* vector of 1s (*N*_*P*_ was number of individuals in training populations); *μ* is the overall mean; *X* is an *N*_*p*_ × *N*_*m*_ matrix of marker genotype indicators (*N*_*M*_ is number of markers in model), with elements *X*_*ij*_ = 0 or 2, if the genotype of line *i* at marker *j* is AA, or BB, respectively; *g* is an *N*_*m*_ × *l* vector of marker breeding value; and *e* is an *N*_*p*_ × *l* vector of residuals.

Estimates of genetic variance (*V*_*g*_) and residual variance (*V*_*e*_) were obtained from an analysis of variance of phenotype within the two IBM populations, and a “mixed population” after combination of both IBM populations. In the following parts, we used *V*_*g*_ and heritability (*h* ^*2*^) estimates from mixed population data (Table 1). The variance of breeding values at each marker locus was assumed to be equal to VgNm, *g* is assumed to be normally distributed, *g*_*i*_ ~ *N* (0, *σ*_*g*_^*2*^).

**Table 1:** Genetic variance (*σ*_*g*_^2^) and heritability (*h* ^*2*^) in different populations.

Population	Number of progenies	Total genetic distance (cM)	GDD		PH	
			*σ*_g_^2^	*h* ^*2*^	*σ*_g_^2^	*h* ^*2*^
Syn10	194	11,198.50	895.96**	0.79	279.18**	0.92
Syn4	244	6,240	1,074.74**	0.81	298.81**	0.90
Mixed population	438	-	1,022.71**	0.80	296.21**	0.90

**Significantly different from zero at the 0.01 level of probability.

### Data analyses and validation

We estimated the marker effects and predicted the genomic breeding values for seven different scenarios (Table 2). We studied the effect of number of markers and also number of progenies in the training population for estimating marker effects. Therefore, we randomly selected seven marker sets with different marker-genome coverage (100, 200, 400, 800, 1600, 3200, and 6611; Table 3) and an even distribution across the genetic map. The number of lines in training populations varied from 30 to 180 with an increment of 30 lines.

**Table 2:** Scenarios for GS tests.

Scenario	Training population	Validation population	Prediction Type
1	Syn10	Syn10	Within population^b^
2	Syn4	Syn4	Within population
3	Syn10	Syn4	Between populations^c^
4	Syn4	Syn10	Between populations
5	Mixed population^a^	Mixed population	Across populations^d^
6	Mixed population	Syn10	Across populations
7	Mixed population	Syn4	Across populations

^a^ Mixed population was a combination of the Syn4 and Syn10 populations

^b^ Lines for training and validation came from the same population

^c^ Lines for training and validation came from different populations

^d^ Lines for training came from both populations.

**Table 3:** Average distance and LD between adjacent markers in different marker sets.

Marker set	Physical Distance	Genetic Distance ^b^	Genetic Distance	LD ^c^ in Syn10	LD in Mixed	LD in Syn4
(*N*_*M*_)	(Mb) ^a^	in Syn4 (cM)	in Syn10 (cM)	(*r* ^*2*^)	Population (*r* ^*2*^)	(*r* ^*2*^)
6,611	0.31	0.94	1.69	0.78	0.81	0.87
3,200	0.64	1.95	3.50	0.66	0.71	0.78
1,600	1.28	3.90	7.00	0.50	0.55	0.63
800	2.57	7.80	14.00	0.32	0.37	0.44
400	5.13	15.60	28.00	0.19	0.22	0.27
200	10.26	31.20	55.99	0.09	0.11	0.15
100	20.53	62.40	111.99	0.05	0.06	0.08

^a^ Average physical distance (in Mb) between adjacent markers.

^b^ Average genetic distance (in cM) between adjacent markers.

^c^ Linkage disequilibrium as estimated by the mean pairwise *r* ^*2*^ values between adjacent markers.

To obtain training populations within a population, lines were randomly selected from the original population. Then the remaining lines were classified as validation population by default. In scenarios 5–7, to obtain training populations from both IBM populations, an equal number of lines were randomly selected from Syn4 and Syn10, respectively. For each scenario (Table 2), sampling of training and validation set was repeated 100 times. The correlation between observed and predicted phenotypes (*r*_*MP*_) was estimated for each repeat. The accuracy of genomic selection was expressed as rMG=rMPh [32,33], where *h* refers to the square root of heritability. Reported prediction accuracies are the mean *r*_*MG*_ values across 100 repeats. Least significant differences (*P* = 0.05, adjusted by the *Bonferroni* procedure for the effect of multiple comparisons) for *r*_*MG*_ were calculated for each scenario, with the combinations of *N*_*P*_ and *N*_*M*_ as independent variables. All data analyses were done in R [34].

## Results

### LD and kinship in two populations

**Fig 1:**
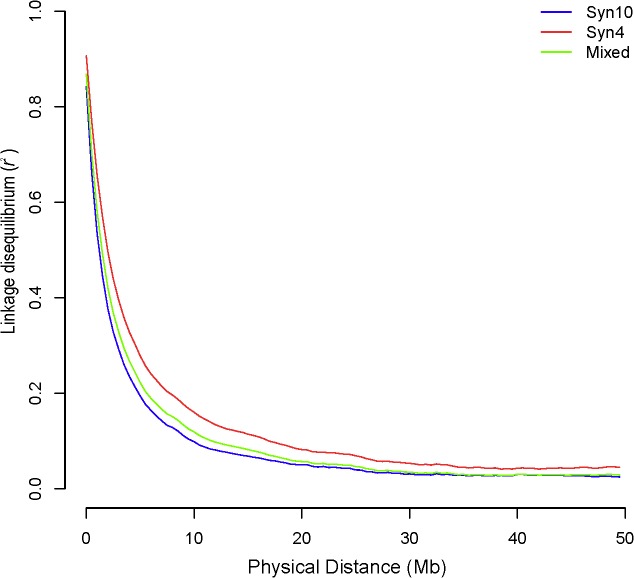
LD decay measured in different populations.

To understand the pattern of LD decay, estimates of pair-wise LD were averaged in increments of 100 kb distance between markers (Fig 1). The average physical distance between adjacent markers varied in different marker sets (Table 1), and average LD between adjacent markers (ALAM) in different marker sets was estimated according to the average distance. Results were listed in Table 3. The ALAM of full marker sets (*N*_*M*_ = 6611) was 0.78, 0.87, and 0.81 in Syn10, Syn4, and mixed population (Table 3), respectively. A high degree of LD was observed even for extended distances between markers. For example, for the marker set of *N*_*M*_ = 800 for Syn10, the average distance between adjacent markers was 2.57 Mb (which equates to 14 cM), but the corresponding LD was 0.32. For marker set of *N*_*M*_ = 400 for Syn4, the average distance between adjacent markers was 5.13 Mb (equal to 15.6 cM), but the corresponding LD was 0.27. As expected, the LD in Syn4 was higher than in Syn10 and in the mixed population. The average realized genetic relationship (kinship) between any progeny pair was 0.63 and 0.44 for Syn4 and Syn10, respectively.

### Trend of prediction accuracies across scenarios

**Fig 2:**
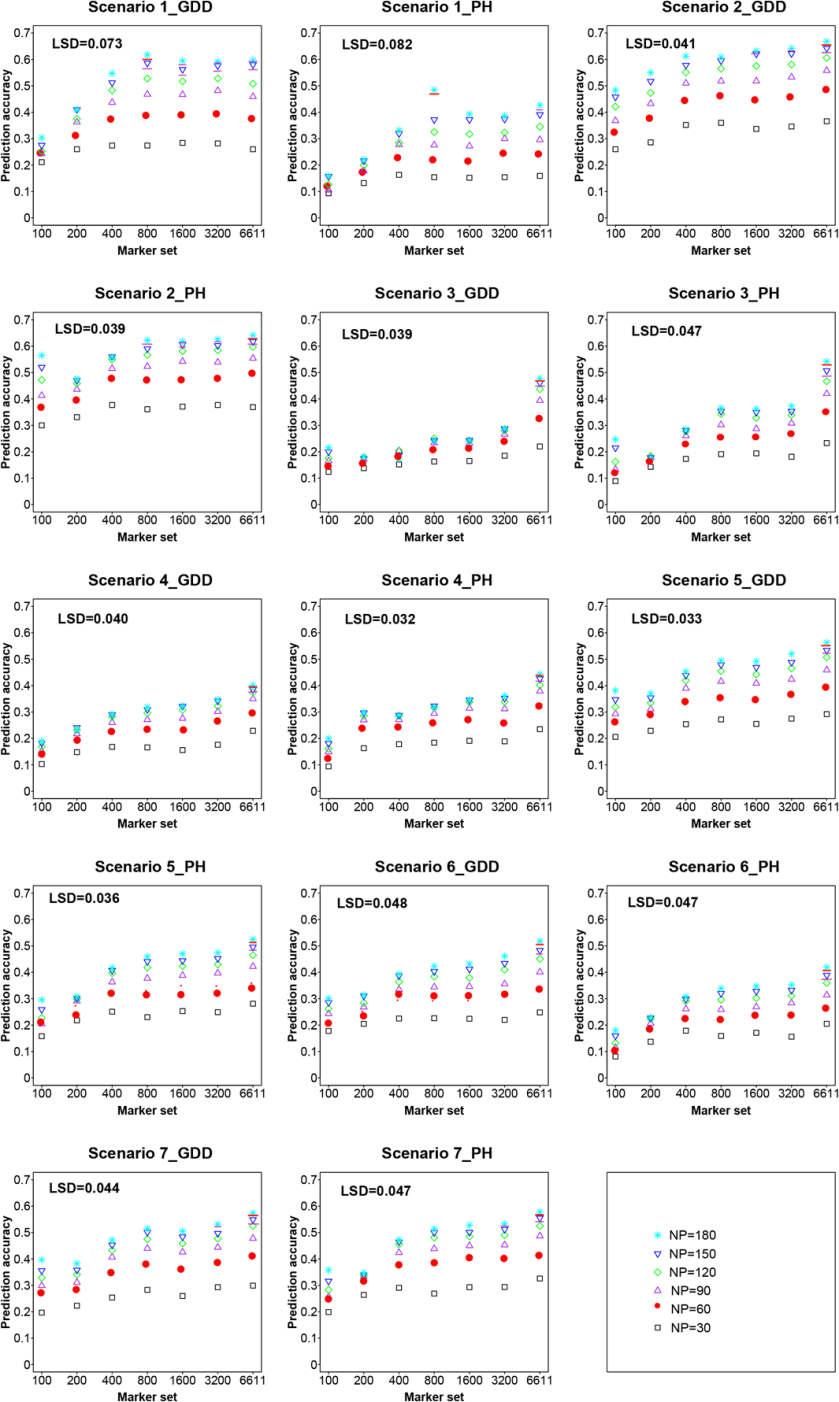
Prediction accuracies depending on marker number (*N*_*M*_) and training population size (*N*_*P*_) for different scenarios and traits.

A nonlinear increase in prediction accuracies with increasing size of *N*_*P*_ and *N*_*M*_ was observed for both traits within both populations (Fig 2, S1 Table). Generally, the highest *r*_*MG*_ values were obtained for the highest *N*_*P*_, and an increase in *N*_*M*_ generally led to increased accuracies. Increase in accuracies was smooth and did not reach an obvious plateau, while increasing *N*_*P*_ using most fixed *N*_*M*_ of all scenarios (S1 Fig). However, the effect of increasing *N*_*M*_ was different under fixed *N*_*P*_ in different scenarios.

### Trend of prediction accuracy within populations

Within populations (Scenario 1 and Scenario 2), accuracy increases became negligible, reached a plateau, or even decreased, when a certain level of *N*_*M*_ was reached. In Scenario 1, when using *N*_*P*_ = 180 for predicting GDD, there was no significant difference in *r*_*MG*_ for *N*_*M*_ from 400–6611. The prediction accuracy for GDD was higher than that for PH (S1 Table). The highest *r*_*MG*_ was obtained (0.49) when *N*_*P*_ = 180 and *N*_*M*_ = 800, although it was not significantly different from *r*_*MG*_ for the highest *N*_*P*_ and *N*_*M*_. In Scenario 2, when marker coverage reached *N*_*M*_ = 1600 (for GDD) or 800 (for PH), *r*_*MG*_ did not significantly increase, if marker density was continually increased. However, when *N*_*P*_ = 180, *r*_*MG*_ for the lowest marker density *N*_*M*_ = 100 could not be ignored. Using *N*_*P*_ = 180, there were no major differences between *r*_*MG*_ of *N*_*M*_ = 100 and *N*_*M*_ = 6611(0.48 *vs*. 0.67 for GDD, 0.57 *vs*. 0.64 for PH). However, in Scenario 2 of PH, *r*_*MG*_ of *N*_*M*_ = 100 was significantly higher compared to *N*_*M*_ = 200, when *N*_*P*_ = 150 (0.52 *vs*. 0.47) or 180 (0.57 *vs*. 0.48) (S1 Table).

### Trend of prediction accuracies between populations

Both in Scenario 3 and Scenario 4, under the same *N*_*P*_, *r*_*MG*_ of *N*_*M*_ = 6611 was significantly higher than for any other marker set, even compared to the next lower marker density *N*_*M*_ = 3200, whereas there was no significant difference between most other adjacent marker densities. However, the average difference between *N*_*M*_ = 6611 and *N*_*M*_ = 3200 under different *N*_*P*_ was more pronounced in Scenario 3 compared to Scenario 4 (0.13 *vs*. 0.05 for GDD, 0.12 *vs*. 0.07 for PH). In Scenario 3, for the highest *N*_*M*_ and *N*_*P*_, *r*_*MG*_ for GDD was 0.48, and *r*_*MG*_ for PH was 0.55. In Scenario 4, for the highest *N*_*M*_ and *N*_*P*_, *r*_*MG*_ for GDD was 0.40, and *r*_*MG*_ for PH was 0.44. In Scenario 4, *r*_*MG*_ was higher than that for Scenario 3 under most different *N*_*M*_, but *r*_*MG*_ of the highest marker sets *N*_*M*_ = 6611 was lower than that for Scenario 3.

### Trend of prediction accuracies for the combined IBM populations

For mixed population (Scenarios 5, 6, and 7), the increased accuracies did not reach a plateau, when increasing *N*_*M*_. Similarly, with the trend of prediction accuracies between populations (Scenarios 3 and 4), *r*_*MG*_ of *N*_*M*_ = 6611 was higher than for any other marker sets, even for the adjacent marker density *N*_*M*_ = 3200 under the same *N*_*P*._ However, not all comparisons showed significant differences. The average difference of *r*_*MG*_ between *N*_*M*_ = 6611 and any other marker set was higher than differences between any of the marker set pairs. For example, in Scenario 5, the average difference of *r*_*MG*_ between *N*_*M*_ = 6611 and 3200 was 0.04 or 0.03, respectively, compared to 0.02 or 0.004 between *N*_*M*_ = 3200 and 1600 for GDD or PH. In these three scenarios, the highest *r*_*MG*_ was found for a combination of the highest *N*_*P*_ and *N*_*M*_. The value of highest *r*_*MG*_ of Scenario 5 was between those for Scenario 6 and Scenario 7 (Scenarios 5, 6, 7 were 0.56, 0.52, 0.57 for GDD, and 0.53, 0.42, 0.58 for PH).

## Discussion

The accuracy of RR-BLUP is due to two main factors, genetic relationship between training and validation individual, and LD between markers and QTL [19,35]. Traditional pedigree-BLUP (Henderson 1975) uses pedigree relationship to determine expected genetic relationship, while RR-BLUP also captures genetic relationships by markers as genomic relationship. Genomic relationships based on genotype data has the capacity to explain actual genetic relationships, deviating from pedigree-based relationships as a consequence of Mendelian sampling [36,37]. Goddard (2009) [38] showed that RR-BLUP is statistically equivalent to a BLUP model, without explicit marker effects and markers only being used to estimate the relationship between lines. Through a simulation study, Habier *et al*. (2007) [19] showed that markers in LE (linkage equilibrium) with QTL could capture genetic relationships, and therefore, affect accuracy of prediction. However, this study also showed that LD between markers and QTL contributed a substantial proportion of the accuracy of RR-BLUP, and accuracy due to LD was more persistent than accuracy due to genetic relationship over generations. Based on the study, we will discuss the effect of genetic relationship within the Syn10 or Syn4 population, respectively, and then explore the potential contribution of LD for prediction accuracy between populations.

### Effect of genetic relationship on prediction accuracy

GS with *r*_*MG*_ of only 0.5 can still result in a two fold higher gain per year compared to traditional MAS in a low-investment wheat breeding program (*h* ^*2*^ = 0.13) and a three fold increase in a high-investment maize breeding program (*h* ^*2*^ = 0.11) [39]. Lorenzana and Bernardo [14] proposed that 100 markers for bi-parental populations and 200–800 markers for random-mated maize populations are sufficient for genome-wide prediction of genotypic values. Using a panel of 788 individuals obtained from a half-diallel cross between four dent inbreeds, [40] showed that marker density is not a major limitation for the accuracy of GS. In scenarios within the two IBM populations (Scenarios 1 and 2), different combinations of *N*_*P*_ and *N*_*M*_ were validated to seek for the lowest marker density to obtain a *r*_*MG*_ comparable to 0.5. In Scenario 2, there was no *r*_*MG*_ in any marker set significantly lower than 0.5, when *N*_*P*_ = 180. One abnormal decrease of *r*_*MG*_ for PH was observed, when the marker density increased from 100 (ALAM = 0.08) to 200 (ALAM = 0.15) (the same phenomenon was observed in Scenario 3). The marker sets of N_*M*_ = 100 and *N*_*M*_ = 200 were randomly and independently selected from the whole marker set. Potentially, more markers of *N*_*M*_ = 100 were in LD with QTL of PH than for *N*_*M*_ = 200 by chance. In Scenario 1 of *N*_*P*_ = 180 and *N*_*M*_ = 100, *r*_*MG*_ of GDD and PH were only 0.304 and 0.159, respectively (compared to 0.48 and 0.57 in Scenario 2), and *r*_*MG*_ exceeded or was near 0.5 until marker density reached 400 (ALAM = 0.19) or 800 (ALAM = 0.32) for GDD or PH, respectively. Thus, more markers were needed in Syn10 to obtain the same accuracy as in Syn4.

The expected (coancestry) relationship within and between pairs of Syn4 and 10 lines does not differ as they derived from the same parents. However, the actual relationships deviated as a consequence of Mendelian sampling. Thus the realized genetic relationship, which is more accurate than expected genetic relationship, was calculated based on markers genotype. The average realized genetic relationship in Syn4 was higher than that in Syn10 (0.63 *vs*. 0.44). Possibly the higher number of generations of intermating in Syn10 reduces average genetic relationship between individuals compared to Syn4.

For *N*_*M*_ = 400 in Scenario 1, the average distance between adjacent markers was 5.1 Mb (28.0 cM) and LD was 0.08 (*r* ^*2*^ = 0.19). For *N*_*M*_ = 100 in Scenario 2, the distance and LD between adjacent markers was 20.5 Mb (62.4 cM) and LD was 0.19 (*r* ^*2*^ = 0.08). Accuracy due to realized genetic relationships can be regarded as lower bounds, if accuracy due to LD is small [22]. Therefore, prediction accuracy in this study was mainly due to realized genetic relationship as the contribution of LD should be limited using low density markers. In previous studies, the accuracy of GS was non-zero, even when the LD between marker and QTL was zero, as the GS model also captures realized genetic relationships [19,35]. However, *r*_*MG*_ of Scenario 2 was higher than that of Scenario 1 at lower marker density. We inferred that more benefits were obtained for related individuals from Syn4 due to higher realized genetic relationships compared to Syn10. This also explains, why the accuracy of Scenario 7 was higher than that of Scenario6. Although both scenarios used mixed populations as training population, the validation populations were from Syn4 and Syn10 for Scenario 7 and Scenario 6, respectively. The difference between the highest *r*_*MG*_ of Scenario 1 and Scenario 2 was 0.05 and 0.18 for GDD and PH, respectively. Similarly, the difference between the highest *r*_*MG*_ of Scenario 6 and Scenario 7 was 0.06 and 0.16 for GDD and PH, respectively. We concluded that both differences were derived from different contributions of realized genetic relationships from Syn4 and Syn10 populations.

In addition to heritability and training population size, the effective number of quantitative trait loci (QTL) was also a factor influencing prediction accuracy [41–43]. Thus, the QTL numbers of two traits were investigated. The result showed that the average number of QTL of GDD for Syn10 and Syn4 was 17.5 and 17 respectively, and the number of QTL of PH for Syn10 and Syn4 was 17 and 16, respectively (unpublished data). The result excluded the effect of number of QTL for prediction accuracy between different Scenarios in this study.

In Scenario 3 and Scenario 4, no *r*_*MG*_ value (correlation between the true genotypic values and the predicted genotypic values based on markers) reached 0.4 when *N*_*M*_ = 800. It seems that the impact of genetic relationship is limited compared to Scenarios within populations. LD should be a good complement in this situation.

### Effect of LD on prediction accuracy

Zhong *et al*. (2009) [18] discussed the effect of LD in RR-BLUP models in another simulation study. According to the study, the dichotomy between contributions “due to LD” *vs*. those “due to genetic relationship” is useful for considering the strengths of different methods, but the two contributions are confounded in practice. Indeed, in most empirical studies, the effect of LD and genetic relationship exist simultaneously. However, the accuracy due to LD may be a lower boundary for the accuracy of an individual that is unrelated to the training population [41]. In our study, due to being derived from the same founders B73 and Mo17, any pair of individuals across two populations was expected to share 50% of the genome. However, two populations have experienced generations of independent intermating. There is no close pedigree relationship between Syn4 and Syn10 lines (other than sharing the same parents), and the genetic relationship between both populations is complicated. This might explain, why Scenarios 3 or 4 did not result in a comparable accuracy at low marker density compared to Scenarios 1 or 2. As Scenarios 3 and 4 remove at least part of these effects due to a close direct genetic relationship, prediction accuracies depends mostly on LD between markers and QTL. It is thus excellent material for studying the effect of LD on prediction accuracy.

In Scenario 3, the training population Syn10 has a lower LD than Syn4. A higher marker density needed to maintain LD between markers and underlying QTL in Syn10 compared to Syn4. Low LD in the training population also means that recombination reduces the size of parental haplotypes, and that the effects of segments can be evaluated more accurately, given sufficient marker coverage. At low marker density in Scenario 3, the prediction accuracy increased slowly with increasing size of the training population. Even at *N*_*M*_ = 3200 (ALAM was 0.66), *r*_*MG*_ of both traits was lower than 0.4. When the marker density was near saturation (*N*_*M*_ = 6611,with ALAM of 0.78), *r*_*MG*_ increased substantially, and reached about 0.5 with *N*_*P*_ = 180. In Scenario 4, the situation was opposite, as the training population Syn4 has a higher LD than Syn10. Low density coverage with markers was sufficient to capture LD, but higher colinearity between adjacent markers (and underlying QTL) hinders evaluation of genome segment effects accurately. Prediction accuracy was higher for Scenario 4 compared to Scenario 3 at low marker coverage, as low density markers also capture part of LD in Scenario 4. With increasing marker density, the *r*_*MG*_ with *N*_*M*_ = 6611 and *M*_*P*_ = 180 was almost 0.1l lower in Scenario 4 than the corresponding *r*_*MG*_ in Scenario 3, as the effect of segments were estimated more accurately in Scenario 3.

In case of a weak genetic relationship between training and breeding population, LD contributed most of the prediction accuracy, thus marker density (LD between adjacent markers) becomes the most important factor. Calus and Veerkamp [44] suggested that an ALAM>0.15 was sufficient for a highly heritable trait. Our study indicated that the contribution of LD for prediction accuracy was limited, when the LD between adjacent markers was only 0.15 (about *N*_*M*_ = 200 for Syn4 in our study). Increasing *N*_*P*_ has almost no effect, until the marker density was high enough to capture LD and to make predictions between 'distant related' populations. We further infer that the prediction accuracy under low marker density is due to other factors, such as genetic relationships.

### The effect of LD and pedigree relationship on GS in maize breeding

A large number of maize DH lines are produced every year. It seems impossible to evaluate all inbred lines for testcross performance in repeated field trials with limited breeding resources. The combination of DH technology and GS revolutionizes maize breeding [45]. Using GS, DH lines can be evaluated based on markers only, thus the cost of genotyping will become the key factor to determine the intensity of application of GS. If, for example, the cost of genotyping on a large scale is 10 cents per data point and the cost of growing a maize yield experimental plot is U.S. $20, genotyping for 200 markers would cost less ($20) than conducting yield trials at three locations ($60) [20]. Costs of genotyping can be expected to drop further, while costs of labor will likely increase (increasing field trial costs) in future.

In our study, a marker density ensuring a LD of 0.1 between adjacent markers was sufficient for acceptable prediction accuracies in bi-parental populations. If training and prediction populations are not closely related, the prediction accuracy will only depend on LD. In this situation, the number of markers required will be larger, a high LD with r^2^>0.8 is needed for acceptable *r*_*MG*_ values. Undoubtedly, this will increase the cost of GS. Therefore, the genetic relationship and LD decay of the population will be of great importance in GS practice.

GS was tested by cross-validation for the Dent heterotic pool, as used on one side of the Flint × Dent pattern in central Europe. Prediction accuracies were near 0.8 for seven biomass- and bioenergy-related traits at a marker density of 5000 (LD between adjacent markers was about 0.1) [21]. The materials from the Dent heterotic pool were highly inbred. Genetic relationships were maintained between “unrelated” lines and could be tracked by genotyping data. In GS application practice, we could genotype and phenotype different populations presenting widely diverse genetic backgrounds to construct a training database. When a new population needs to be evaluated by GS, we can select the best training set from a training database according to the genetic relationship between prediction population and potential training set, to reduce the marker density requirement for prediction.

LD is an important factor that needs to be considered, when there is only a weak genetic relationship for prediction. In Scenarios 3 and 4, the impact of LD in the training population was observed. High LD in the training population not only means a lower marker density requirement to cover the genome, but also means a higher colinearity between linked markers, hindering evaluation of small genome segment effects accurately. This will reduce the robustness of prediction models for other populations. Therefore, if the prediction model is used temporarily, a training population with high LD is suggested for lower marker density. But if the prediction model is very important and will be used frequently, a low LD training population is recommended for extensive and persistent fitness over generations. A saturated marker density is suggested for such training populations to obtain an accurate and robust prediction model. The marker density for the prediction population will be determined according to LD in the prediction population, and the process of imputation (such as for Syn4 in our study) will help to ensure consistent marker sets between training and prediction populations to obtain accurate prediction.

In our study, Syn4 and Syn10 were derived from the same parents and under high levels of identity by descent, however they didn’t predict each other well. While the reason related to genetic relationship and LD was discussed above, there were two more potential factors that shouldn’t be ignored. One was the genotyping error in syn10 and syn4 populations. We planned to randomly extract parts of the lines from two populations and validate their genotype by restriction-site associated DNA tags (RAD) sequencing. The other potential factor was that two populations grew in adjacent locations, which might induce extra environmental error. In the two populations, limited genetic diversity and clear genetic background help us simplify the analysis and draw a preliminary conclusion. However, the shortcoming of our study is also obvious. Diverse materials were used in practical breeding program, and several issues should be solved for application of genomics selection. For instance, lower identity by descent, faster LD decay, and three or more genotypes in one locus due to high genetic diversity. Next step, we will gradually extend our study to more diverse materials, so as to further study the impact of genetic relationship and LD in practice.

## Supporting Information

S1 Table Prediction accuracy (*r*_*MG*_) obtained from the different combinations of *N*_*P*_ and *N*_*M*._
(XLSX)

S1 Fig Prediction accuracy with increasing *N*_*P*_ using fixed *N*_*M*._
(TIFF)
