# Correction: The Impact of Genetic Relationship and Linkage Disequilibrium on Genomic Selection

**DOI:** 10.1371/journal.pone.0162057

**Published:** 2016-08-25

**Authors:** Hongjun Liu, Huangkai Zhou, Yongsheng Wu, Xiao Li, Jing Zhao, Tao Zuo, Xuan Zhang, Yongzhong Zhang, Sisi Liu, Yaou Shen, Haijian Lin, Zhiming Zhang, Victor Abertondo, Michael Lee, Kaijian Huang, Thomas Lübberstedt, Guangtang Pan

Dr. Victor Abertondo and Dr. Michael Lee are not included in the author byline. They should be listed as the thirteenth and fourteenth authors respectively and are both affiliated with the Department of Agronomy, Iowa State University, Ames, Iowa, United States of America. The contributions of Dr. Victor Abertondo are as follows: Conceived and designed the experiments, performed the experiments, and analyzed the data. The contributions of Dr. Michael Lee are as follows: Conceived and designed the experiments, performed the experiments, analyzed the data, contributed reagents/materials/analysis tools, and wrote the manuscript.

## References

[1] Liu H, Zhou H, Wu Y, Li X, Zhao J, Zuo T, et al (2015) The Impact of Genetic Relationship and Linkage Disequilibrium on Genomic Selection. PLoS ONE 10(7): e0132379 doi: 10.1371/journal.pone.0132379 26148055 10.1371/journal.pone.0132379 PMC4493124

