# Supplementary figures and images for: The Impact of Genetic Relationship and Linkage Disequilibrium on Genomic Selection
Source: PLoS One. 2015 Jul 6;10(7):e0132379. doi: 10.1371/journal.pone.0132379 (PMC4493124; doi:10.1371/journal.pone.0132379)

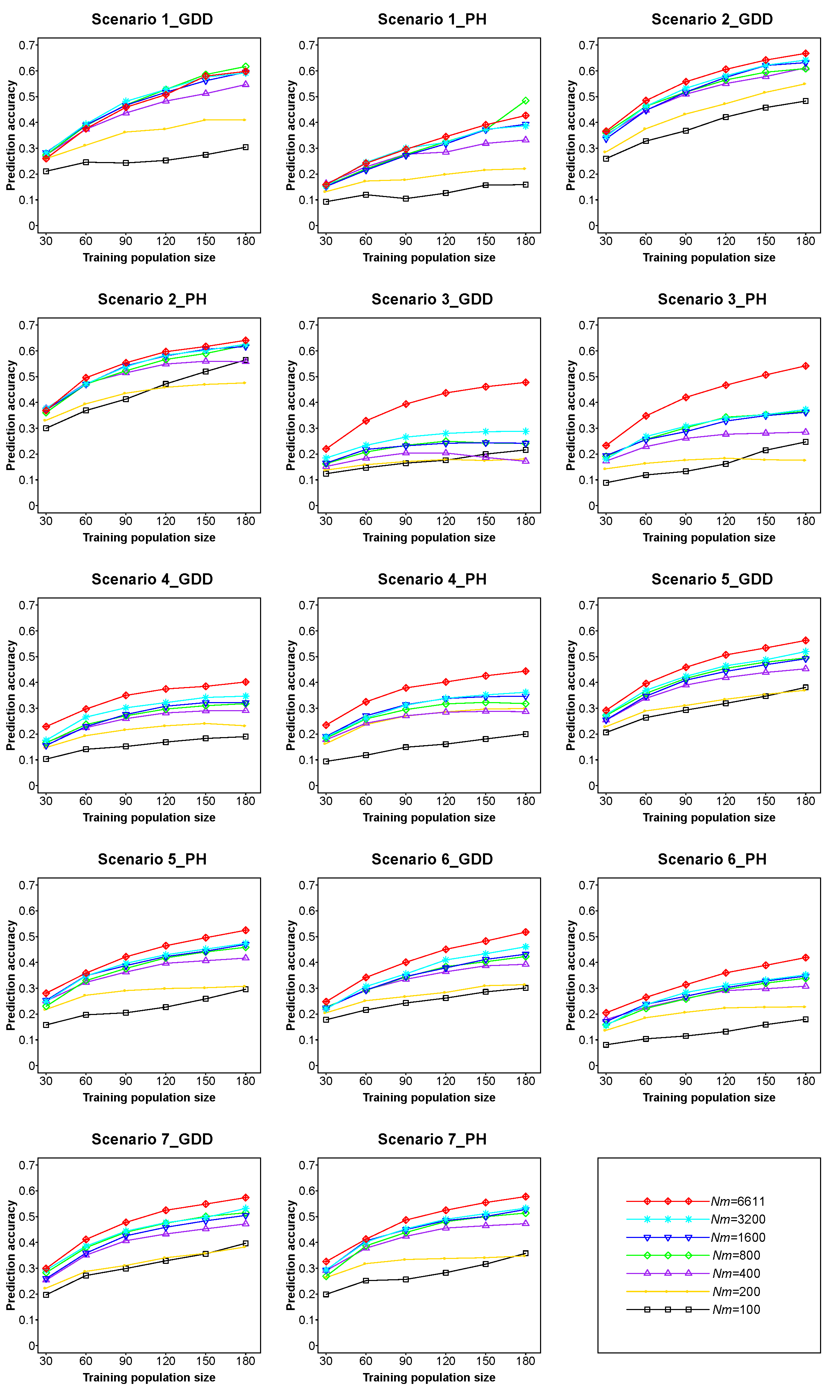

Supplement: S1 Fig — (TIFF) [file pone.0132379.s002.tiff]
